# Supplementary material for: Superiority of Cellulose Non-Solvent Chemical Modification over Solvent-Involving Treatment: Solution for Green Chemistry (Part I)
Source: Materials (Basel). 2020 Jun 3;13(11):2552. doi: 10.3390/ma13112552 (PMC7321458; doi:10.3390/ma13112552)
Supplement: Supplementary file 1 [file materials-13-02552-s001.pdf]

materials

Article

Superiority of Cellulose Non-Solvent Chemical Modification over Solvent-Involving Treatment: Solution for Green Chemistry (Part I)

Stefan Cichosz and Anna Masek *

Lodz University of Technology, Institute of Polymer and Dye Technology, Faculty of Chemistry, 90-924 Lodz, Stefanowskiego 12/16, Poland; stefan.cichosz@dokt.p.lodz.pl
* Correspondence: anna.masek@p.lodz.pl

Received: 28 April 2020; Accepted: 01 June 2020; Published: date

1. Method

1.1. Near Infrared Spectroscopy (NIR)

In this method, the near-infrared region of the electromagnetic spectrum was used. The measurements were carried out in the range of 10000–4000 cm^{-1} in absorption mode—64 scans (Thermo Scientific, Nicolet 6700). Cellulose fibres were dried for 24 h at 100 °C (Binder® oven) before being analysed.

2. Near Infrared Spectra Analysis

The modification process was also tracked with the employment of NIR technique, which according to the literure, is more sensitive to polar groups [1–3]. Nevertheless, this method is not widely-known, but it seems a perfect tool for cellulose structure [4] and moisture content [5] investigation.

Considering the NIR spectra of cellulose fibres (Figure S1) some signals characteristic of this substance can be recognized, e.g., 8165 cm^{-1} (C–H stretching of the second overtone) [6], 7290 cm^{-1} (C–H stretching and C–H deformation vibrations) [6], 6472 cm^{-1} (O–H stretching of the first overtone) [6], 4754 cm^{-1} (O–H stretching vibrations) [7]. Absorption bands assigned to chemical groups typical for cellulose fibres are tabularized in Table S1.

Regarding Figure S1 it may be evidenced again that the modification process of cellulose fibres with silane coupling agents causes some shifts between the peaks visible in the spectra. The shifts are stronger for absorption bands assigned to the hydroxyl groups through which the coupling occurs. This may be an important observation considering the effect of the treatment process on the chemical structure of the analysed cellulose fibres.

Furthermore, while having a closer look at the data visible in Figure S1b, one could easily observe that the shift of the absorption band maximum at 6715 cm^{-1} is stronger in comparison with the same shift visible in Figure S1a. This confirms the interpretation of results presented in the previous section—cellulose treated via a non-solvent method in a planetary mill is possibly modified in a more efficient way.

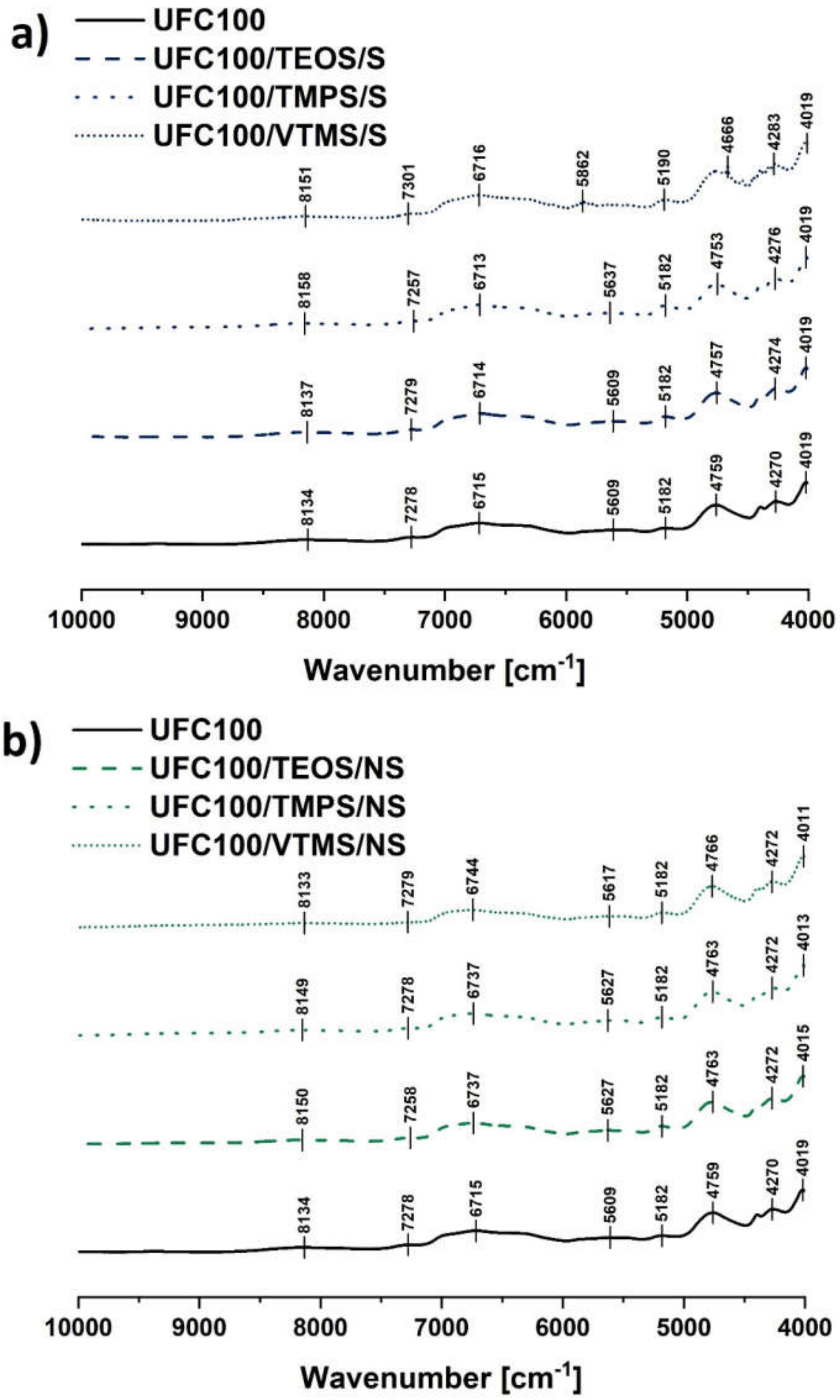

Figure S1. Near infrared spectra of: (**a**) cellulose fibres modified via a solvent-involving method; (**b**) cellulose fibres modified via a non-solvent method. Characteristic absorption bands: 8200–8100 cm^{-1} (C–H), 6770 cm^{-1} (O–H), 6715 cm^{-1} (O–H, water), 4750 cm^{-1} (O–H).

Table S1. Tabularized absorption bands assigned to the chemical groups.

Wavenumber [cm^{-1}]	Chemical Group	Ref.
4750	–OH, C=O	[7]

5220–5150	–OH, water	[6]
6715	–OH, water, hydrogen bonds	[8]
6770	–OH	[9]
8200–8100	C–H	[6]

Yet, it should be considered that intensity differences described above could also be caused by moisture content variations. Therefore, they may only indicate the possibility of hydroxyl moiety amount decrease but cannot be considered as evidence for the modification process occurring.

Moreover, taking into consideration the intensity differences of signals assigned to hydroxyl groups, which are present in the given spectra, it may be said that blocking of –OH moieties of the cellulose chains by silanes coupled to the UFC100 surface, occurs to a varied extent and the reactivity of the modifying agents is not the same. This phenomenon has been evidenced in the literature previously—linear silane compounds with methoxyl groups are referred to as being the most suitable for the conducted reaction with cellulose fibres [10].

It is advised more to analyse the shifts between the peaks as they are less affected by the water content [11,12].

Summarising, NIR analysis enabled confirmation of the structural changes visible in the FT-IR spectra. Shifts between the peaks are stronger in the case of samples modified via a non-solvent approach which may indicate some variations in chemical composition of analysed cellulose fibres, e.g.,–OH blocking, decreased moisture content.

References

1. Ambjörnsson, H.A.; Schenzel, K.; Germgård, U. Carboxymethyl cellulose produced at different mercerization conditions and characterized by nir ft raman spectroscopy in combination with multivariate analytical methods. *BioResources* **2013**, *8*, 1918–1932.
2. Schenzel, A.; Hufendiek, A.; Barner-Kowollik, C.; Meier, M.A.R. Catalytic transesterification of cellulose in ionic liquids: Sustainable access to cellulose esters. *Green Chem.* **2014**, *16*, 3266–3271.
3. He, W.; Hu, H. Prediction of hot-water-soluble extractive, pentosan and cellulose content of various wood species using FT-NIR spectroscopy. *Bioresour. Technol.* **2013**, *140*, 299–305.
4. Delwiche, S.R.; Pitt, R.E.; Norris, K.H. Examination of Starch-Water and Cellulose-Water Interactions With Near Infrared (NIR) Diffuse Reflectance Spectroscopy. *Starch - Stärke* **1991**, *43*, 415–422.
5. Cichosz, S.; Masek, A. Cellulose Fibers Hydrophobization via a Hybrid Chemical Modification. *Polymers (Basel).* **2019**, *11*, 1174.
6. Schwanninger, M.; Rodrigues, J.C.; Fackler, K. A review of band assignments in near infrared spectra of wood and wood components. *J. Near Infrared Spectrosc.* **2011**, *19*, 287–308.
7. Ali, M.; Emsley, A.M.; Herman, H.; Heywood, R.J. Spectroscopic studies of the ageing of cellulosic paper. *Polymer (Guildf).* **2001**, *42*, 2893–2900.
8. Inagaki, T.; Siesler, H.W.; Mitsui, K.; Tsuchikawa, S. Difference of the crystal structure of cellulose in wood after hydrothermal and aging degradation: A NIR spectroscopy and XRD study. *Biomacromolecules* **2010**, *11*, 2300–2305.
9. Rantanen, J.; Räsänen, E.; Tenhunen, J.; Känsäkoski, M.; Mannermaa, J.P.; Yliruusi, J. In-line moisture measurement during granulation with a four-wavelength near infrared sensor: An evaluation of particle size and binder effects. *Eur. J. Pharm. Biopharm.* **2000**, *50*, 271–276.
10. Nakatani, H.; Iwakura, K.; Miyazaki, K.; Okazaki, N.; Terano, M. Effect of Chemical Structure of Silane Coupling Agent on Interface Adhesion Properties of Syndiotactic Polypropylene/Cellulose Composite. *J. Appl. Polym. Sci.* **2011**, *119*, 1732–1741.
11. Hofstetter, K.; Hinterstoisser, B.; Salmén, L. Moisture uptake in native cellulose - The roles of different hydrogen bonds: A dynamic FT-IR study using Deuterium exchange. *Cellulose* **2006**, *13*, 131–145.
12. Loof, D.; Hiller, M.; Oschkinat, H.; Koschek, K. Quantitative and qualitative analysis of surface modified cellulose utilizing TGA-MS. *Materials (Basel).* **2016**, *9*, 1–14.

© 2020 by the authors. Licensee MDPI, Basel, Switzerland. This article is an open access article distributed under the terms and conditions of the Creative Commons Attribution (CC BY) license (http://creativecommons.org/licenses/by/4.0/).
